# Editorial: Sleep health and measures

**DOI:** 10.3389/fpubh.2024.1476077

**Published:** 2024-09-13

**Authors:** Runtang Meng, Haiyan Ma, Karen Spruyt, Esther Yuet Ying Lau, Joseph M. Dzierzewski

**Affiliations:** ^1^School of Public Health, Hangzhou Normal University, Hangzhou, Zhejiang, China; ^2^Engineering Research Center of Mobile Health Management System, Ministry of Education, Hangzhou, Zhejiang, China; ^3^Université Paris Cité, NeuroDiderot, INSERM, Paris, France; ^4^Sleep Laboratory, Department of Psychology, The Education University of Hong Kong, Hong Kong, Hong Kong SAR, China; ^5^Centre for Psychosocial Health, The Education University of Hong Kong, Hong Kong, Hong Kong SAR, China; ^6^Centre for Religious and Spirituality Education, The Education University of Hong Kong, Hong Kong, Hong Kong SAR, China; ^7^National Sleep Foundation, Washington, DC, United States

**Keywords:** sleep health, measures, quality of life, wellbeing, population health

In the past few decades, studies on sleep have been increasing in number, as an important crossroads of different disciplines including psychiatry, neurology, neuroscience, respiratory medicine, psychology, nursing, and public health (1, 2). Moving beyond studies of basic sleep science and sleep disorders, sleep health has emerged as a new direction that provides a more holistic framework for the main dimensions of sleep: regularity, satisfaction, alertness, timing, efficiency, and duration (1, 3). Given its growing importance, it is imperative to evaluate, consolidate, and further develop measures of sleep health so that the global sleep research community has more valid tools to deploy.

A growing body of research has revealed that poor sleep health is associated with poor health behaviors (e.g., smoking and alcohol consumption), negative psychological states (e.g., anxiety, depression, and perceived stress), chronic diseases (e.g., cardiovascular disease and diabetes), and physical injuries (e.g., road traffic injuries). Irregular sleep schedules, bedtime procrastination, and other unhealthy sleep behaviors such as widespread use of electronic devices and social media, may negatively impact sleep health in modern societies (4). As good sleep health is a critical cornerstone for overall health, routine monitoring, multi-level preventive measures, and interventions are needed to further elucidate an unmet public health problem and improve health equity (5).

To this end, our Research Topic “*Sleep health and measures*” includes original research articles, reviews, and opinion pieces that explore the current state of sleep health, its influencing factors, and key health outcomes. By identifying approaches to accurately measure, assess, and improve sleep health, the current Research Topic strives to further substantiate the significance of sleep health as a multidisciplinary field of research. In this editorial, we provide a brief overview of selected articles published on the Research Topic “*Sleep health and measures*” in the journal *Frontiers in Public Health* (https://www.frontiersin.org/research-topics/48787/sleep-health-and-measures/articles).

The study by Tesfaye et al. aimed to determine the prevalence and associated factors of poor sleep quality among healthcare professionals in Northwest Ethiopia. Using a cross-sectional design and stratified random sampling, data were collected from 418 participants using self-administered questionnaires. Results revealed a high prevalence of poor sleep quality (58.9%). Factors significantly associated with poor sleep quality included being female, shift work, a lack of regular exercise, khat chewing, and so on. The authors recommend early screening for sleep disturbances, better management of shift work schedules, and interventions targeting lifestyle behaviors and mental health support, to improve sleep quality among healthcare professionals.

Guo et al. investigated how sleep quality, perceived stress, and social support interact to influence depression in stroke patients. Conducted between January and May 2023 using cluster random sampling in five hospitals in Henan Province, China, this multicenter cross-sectional survey with 471 stroke patients revealed that poor sleep quality partially mediated the relationship between perceived stress and depression. Moreover, social support moderated this mediation effect. These findings suggest that improving sleep quality and social support may be key strategies to mitigate depression in stroke patients, providing a foundation for developing targeted intervention programs.

The study by Loke et al. reported on the development and validation of the Sleep Health And Wellness Questionnaire (SHAWQ) to identify sleep problems and potential depression risks in adolescents and university students. Using a 6-item questionnaire derived from key sleep and health predictors, the SHAWQ was tested in four studies involving 8,567 adolescents and university students. The tool demonstrated good convergent validity, test-retest reliability, and predictive validity (for academic performance), indicating that the SHAWQ is effective for screening for sleep health and mood issues in teenagers and young adults, which could guide interventions to improve sleep and mental health status in this at-risk segment of the population.

The systematic review by González-Martín et al. was an instrumental addition to this Research Topic. This meta-analysis evaluated the effectiveness of mindfulness-based programs on sleep quality in healthy, non-institutionalized older adults. Following PRISMA guidelines, the review included 10 out of 177 initially identified articles from four databases (Pubmed, Scopus, Web of Science, and CINAHL) searched in May and June 2023. Overall, older adults who underwent mindfulness-based programs indicated significant improvements in sleep quality, with moderate effect sizes across different sleep assessment tools. The findings suggest that mindfulness may be an effective treatment for insomnia symptoms and other sleep quality issues in older adults.

The breadth of measurements, measurement strategies, and related health outcomes investigated in the articles included in this Research Topic, highlights not only the excitement surrounding sleep health, but also the challenges inherent in any emerging area of investigation. We encourage scholars to be intentional in their selection of both measures and outcomes. As the accumulation of knowledge is dependent on the ability to synthesize across studies, much progress in our understanding of sleep health can be made with small advances in conceptualization and measurement.
